# Accuracy of guide wire placement for femoral neck stabilization using 3D printed drill guides

**DOI:** 10.1186/s41205-022-00146-8

**Published:** 2022-07-04

**Authors:** Gregory R. Roytman, Alim F. Ramji, Brian Beitler, Brad Yoo, Michael P. Leslie, Michael Baumgaertner, Steven Tommasini, Daniel H. Wiznia

**Affiliations:** 1 Orthopaedics and Rehabilitation, Yale School of Medicine, Yale University, New Haven, CT USA; 2 Yale Center for Medical Informatics, Yale School of Medicine, Yale University, New Haven, CT USA; 3 VA Connecticut Healthcare System, Veterans Health Administration, West Haven, CT USA; 4 Biomedical Engineering, Yale School of Engineering and Applied Science, Yale University, New Haven, CT USA; 5 Mechanical Engineering & Materials Science, Yale School of Engineering and Applied Science, Yale University, New Haven, CT USA

**Keywords:** 3D printing, Drill guide, Prophylaxis, In silico, Percutaneous screws, Femoral neck system, Dynamic hip screw

## Abstract

**Background:**

The goal of stabilization of the femoral neck is to limit morbidity and mortality from fracture. Of three potential methods of fixation, (three percutaneous screws, the Synthes Femoral Neck System, and a dynamic hip screw), each requires guide wire positioning of the implant(s) in the femoral neck and head. Consistent and accurate positioning of these systems is paramount to reduce surgical times, stabilize fractures effectively, and reduce complications. To help expedite surgery and achieve ideal implant positioning in the geriatric population, we have developed and validated a surgical planning methodology using 3D modelling and printing technology.

**Methods:**

Using image processing software, 3D surgical models were generated placing guide wires in a virtual model of an osteoporotic proximal femur sawbone. Three unique drill guides were created to achieve the optimal position for implant placement for each of the three different implant systems, and the guides were 3D printed. Subsequently, a trauma fellowship trained orthopedic surgeon used the 3D printed guides to position 2.8 mm diameter drill bit tipped guide wires into five osteoporotic sawbones for each of the three systems (fifteen sawbones total). Computed Tomography (CT) scans were then taken of each of the sawbones with the implants in place. 3D model renderings of the CT scans were created using image processing techniques and the displacement and angular deviations at guide wire entry to the optimal sawbone model were measured.

**Results:**

Across all three percutaneous screw guide wires, the average displacement was 3.19 ± 0.12 mm and the average angular deviation was 4.10 ± 0.17^o^. The Femoral Neck System guide wires had an average displacement of 1.59 ± 0.18 mm and average angular deviation of 2.81 ± 0.64^o^. The Dynamic Hip Screw had an average displacement of 1.03 ± 0.19 mm and average angular deviation of 2.59 ± 0.39^o^.

**Conclusion:**

The use of custom 3D printed drill guides to assist with the positioning of guide wires proved to be accurate for each of the three types of surgical strategies. Guides which are used to place more than 1 guide wire may have lower positional accuracy, as the guide may shift during multiple wire insertions. We believe that personalized point of care drill guides provide an accurate intraoperative method for positioning implants into the femoral neck.

## Introduction

As the elderly population increases worldwide, the number of hip fractures is expected to rise to between 6.1 million and 8.2 million by 2050 [1]. The need for stabilization of hip fractures, and particularly the femoral neck, has therefore become an important and useful tool which can help patients whose fractures can be managed using osteosynthesis. Varying surgical strategies are constrained by the dimensions of equipment and the precision which is required to make these procedures successful and tailored to their patients’ needs [2, 3]. In a study by Kain et al. [2], implants failed in part due to malpositioning, with 10% of patients requiring total hip arthroplasty within 9 months post-operation. Precision and accuracy are thereby crucial factors in femoral neck fracture stabilization.

With the advent of high resolution imaging technology, custom orthopedic surgical instruments has been rendered possible and has been widely published [4–6]. The process of 3D printing has opened new horizons for this personalization of medicine to become a reality, shortening surgical times and reducing operative errors [7–10]. These solutions use a patient’s own anatomy, as imaged in Computed Tomography (CT) scans or MRI to create personalized surgical instruments to perform a custom procedure. Traditional instruments attempt to be universal and do not take into consideration unique anatomical shapes and forms. However, 3D printing of instruments provides the ability to rapidly construct personalized tailored solutions. In a hip surface replacement study by Raaijmaakers [11], for instance, the team constructed patient specific CT based femoral neck drill guide devices. The eventual placement of the devices was rated by surgeons as one which was minimally complex and was accurately placed according to the preoperative surgical plan [11]. Our research group has been studying the use of implants to provide stabilization of femoral neck fractures [12]. Through an iterative process of drill guide design, our group’s goal was to design an accurate and personalized solution to help expedite and improve the accuracy of fixation of the femoral neck. We hope to expand this work to cadaveric models and eventually clinical trials in human subjects.

**Fig. 1:**
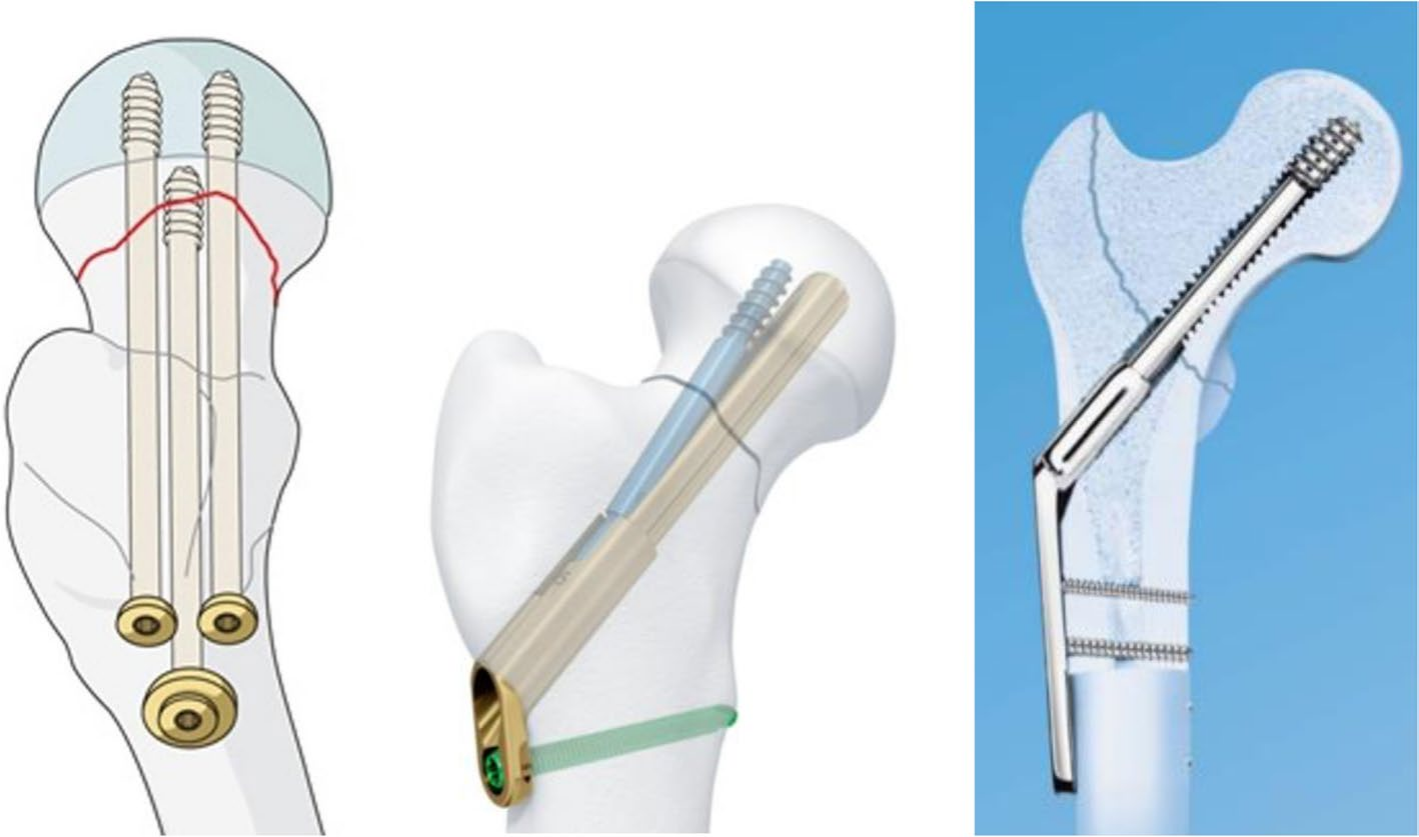
Three possible methods of femoral neck stabilization: percutaneous screws (left) [12] Synthes Femoral Neck System (FNS) (middle) [13], and dynamic hip screw (DHS) (right) [14]

We aim to validate the accuracy of custom 3D printed drill guides for percutaneous screws, the Synthes Femoral Neck System (FNS), and dynamic hip screw (DHS) (Fig. 1). The accuracy of these models will provide robust application of guide wires for increased confidence of placement based on preoperative CT images and leveraging 3D printing technology for manufacturing point of care pre-surgical instruments.

## Methods

### Creation of drill guides

Using Synopsys Simpleware ScanIP image processing software, the three implant systems (three percutaneous screws, FNS, and DHS), were positioned and implanted in consultation with two fellowship trained orthopedic trauma surgeons. The surgical technique stipulated that the FNS system’s drill guide path was to be angulated at 130^o^ and the DHS system’s drill guide path was to be angulated at 135^o^ relative to the lateral border of the femoral diaphysis. Implant CAD files were provided by the manufacturer (Depuy Synthes). Guide wires were modeled after 2.8 mm diameter guide wires across all systems. The guide wires were simulated in ScanIP by creating primitive cylindrical geometries of height 120 mm and diameter 40 mm. Drill guides were created with 3.2 mm holes to provide for additional space for the wire to slide through, while limiting toggle to control for accurate placement. The path for each guide wire to traverse through in each of the drill guides was made a uniform 2 cm. All drill guides had the conformational shape of an osteoporotic sawbone (Osteoporotic Femur, Composite, 10 PCF Solid Foam with 16 mm Canal, Medium) subtracted from it, so that the custom drill guide would sit on the sawbone. These drill guides were printed using the Form3 3D Printer from FormLabs®. This inverted vat polymerization allows for both quick and efficient printing, which is ideal for scaling this system up to eventual regular clinical use, and allows for a high level of detail which allows for improved personalization of drill guides to patients’ anatomy. Printing conformation was designed so that internal supports attached to the guides from their external surfaces, so that removal of the supports did not leave residue which might have otherwise obstructed conformation onto the osteoporotic sawbone. They were created using Grey V4 resin and post-processed according to manufacturer guidelines. This resin, although not biocompatible like other resins produced by FormLabs®, provided both the provided both the proper flexibility to conform to the sawbone femur and stiffness to provide a path for the guide wire that was needed for this sawbone model. The printed guides were then washed for 10 minutes each in isopropyl alcohol and cured for 30 minutes each.

### Drilling osteoporotic sawbones with 3D printed drill guides

The fellowship trained orthopedic surgeon drilled the 2.8 mm guide wires with standard point drill tips. The surgeon used a technique of positioning the drill guide to its most stable conformation on the osteoporotic sawbones and drilled with a Stryker System 7 wire driver power system using a peck drill technique. The drill guides were then removed, the wires were retained, and the osteoporotic sawbone femurs were scanned with a LightSpeed VCT GE Medical Systems Computed Tomography (CT) Scanner using a slice thickness of 0.625 mm and 80 kVp.

### Design iterations

**Fig. 2:**
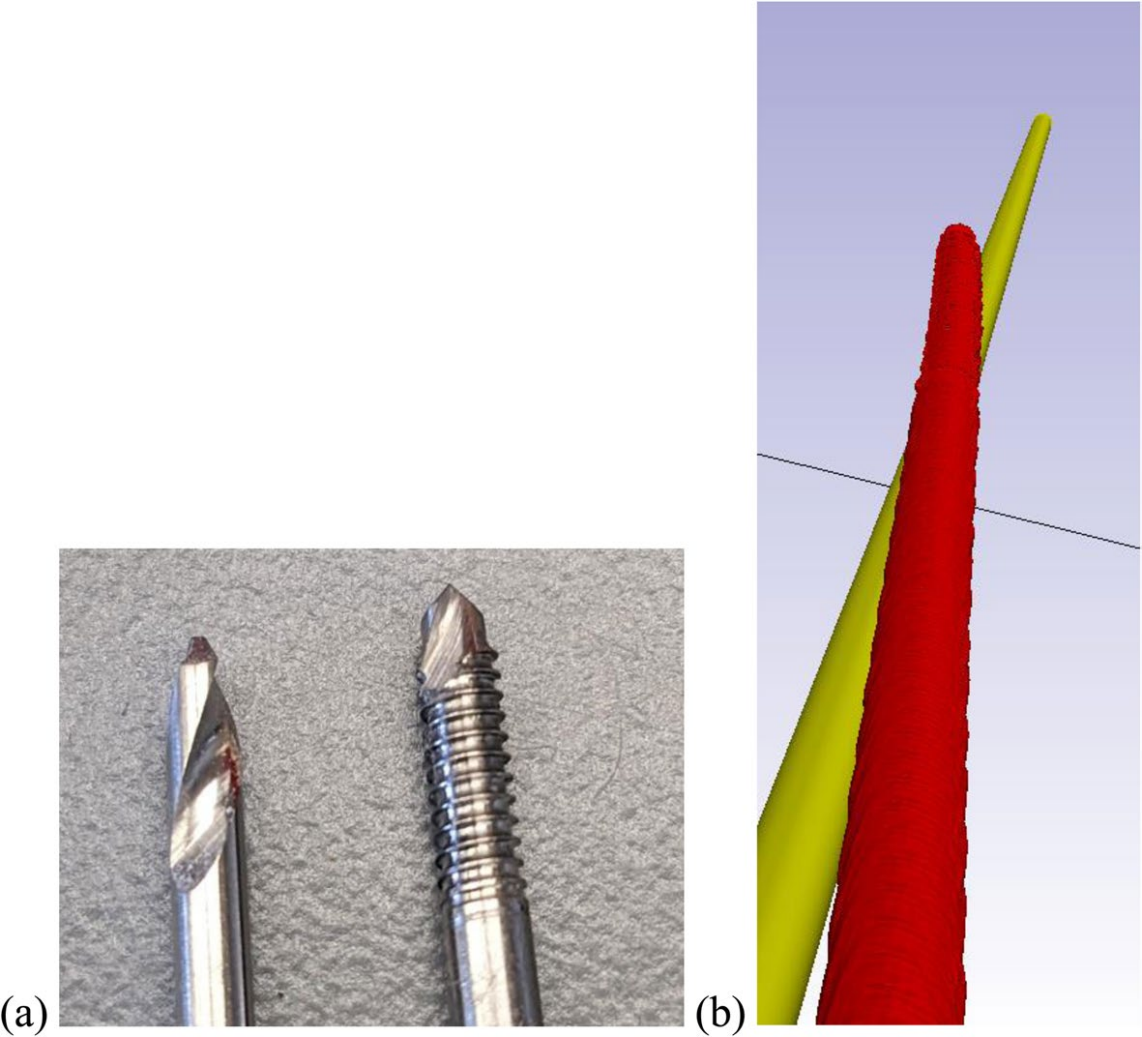
**a** Comparison of threaded tip guide wire (right) vs drill-bit tip guide wire (left). **b** Bending of thread-tip wire in the femur model during the second iteration of the drill guide with the ideal (gold) and experimental (red) paths traversed by the threaded tip guide wire shown

We had multiple design iterations to improve the drill guides, in which we adjusted the placement of the guide onto the sawbone femur and modified features such as the channels for the guide wires (Table 1). In addition, we determined that a drill-bit tip was superior to a threaded tip to prevent slipping on the cortex and deviation of the drill path within the sawbone (Fig. 2). The drill-bit tip aids in preventing the guide wire deviating from its designed path (CT scanned red guide wire in Fig. 2b) by cutting a path, while the threaded tipped wire pulls through the material and may deviate by following a path of least resistance. In addition, we utilized the technique of peck-drilling, in which drill bit tipped guidewire was advanced a depth of no more than five times the diameter of the drill before retracting it to the surface to clean the cutting flutes.

**Table 1:** Iterations of 3D printed drill guide being used to drill osteoporotic sawbone femurs

Iteration	Description	Figure
1	All models were created with a cylindrical shell, just covering the lower half of the greater trochanter. Additional coverage of the greater trochanter was needed. Tubes leading from guide wire entry point to sawbone cortex are external.	
2	Tubes were lengthened, however proved too flexible and more prone to breakage.	
3	Internal channels were created to negate the effects of the excessively flexible tubes. However, the channel was too narrow to provide a consistent trajectory (too much toggle)	
4	A guide was made using Formlabs Rigid Resin. However, the highly stiff material did not allow for flexible conformation to the sawbones.	
5	The Percutaneous Screw Guide had a slightly lowered turret to minimize cortical breach in the femoral neck superiorly. Channel length of 2 cm was standardized across all models. The guides are shown left to right: Percutaneous Screw Guide, FNS Guide, DHS Guide.

### Comparison of drilled sawbones with simulation of guide wires

**Fig. 3:**
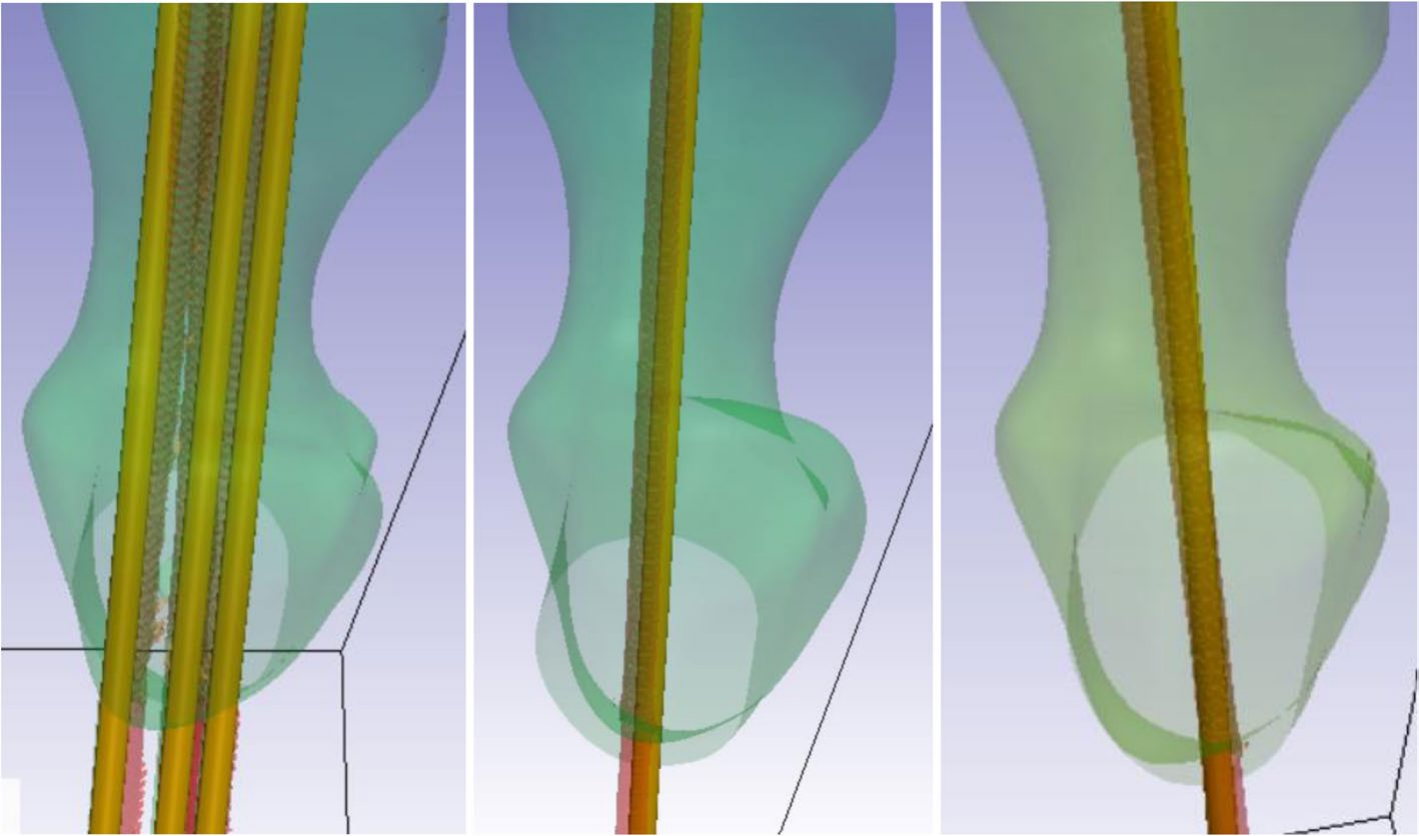
3D overlay of expected model over CT-scan generated mask simulations. Green structures are the femur, gold are the ideal guide wires generated by the 3D modelling software, and the red structures are actual wires from 3D generated models based on overlayed CT scans (left to right: Percutaneous Screw Guide, FNS Guide, DHS Guide). Drill-bit tip guide wires were used in all scenarios

Using ScanIP image processing software, the CT scans of guide wires and sawbone femurs were rendered as 3D model masks (Fig. 3). The preoperative planned guide wires and sawbones were then overlayed over these CT models. Displacement and angular deviation of the CT guide wires was measured at the cortical entry point of the guide wires using ScanIP’s measurement tool. Displacement and angular deviation were recorded over the three system scenarios, five sawbone femurs each – totaling 25 guide wire placements.

### Statistical analysis

**Fig. 4:**
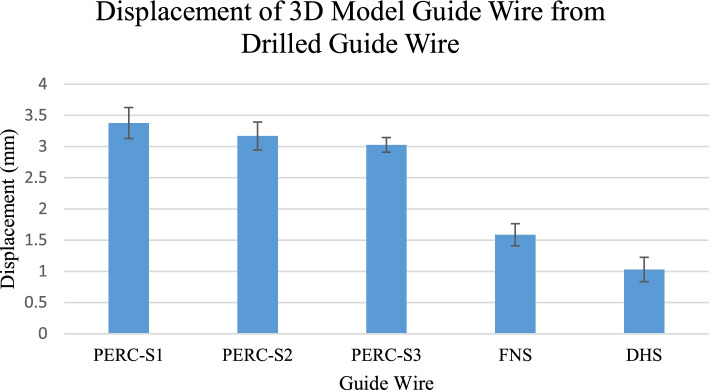
Displacement of 3D model guide wire from physically drilled guide wire. Through ANOVA and t-test statistical analysis, we determined that PERC-S1, PERC-S2, and PERC-S3 were significantly higher in displacement compared with FNS and DHS

**Fig. 5:**
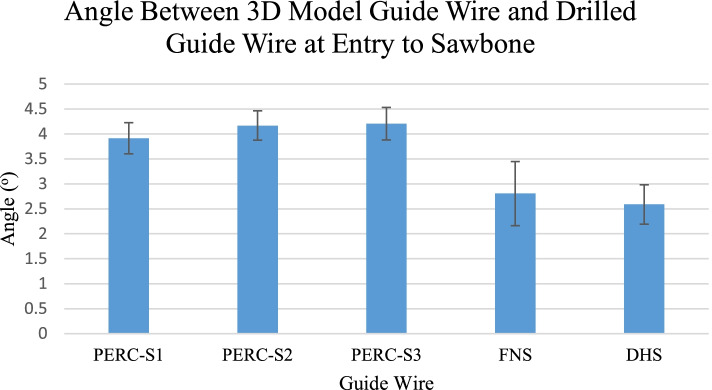
Angle between 3D model guide wire and drilled guide wire at entry to sawbone. Through ANOVA and t-test statistical analysis, we determined that the guide for the DHS was significantly lower in angular deviation compared to the guide for the percutaneous screws

For both displacement (Fig. 4) and angular deviation (Fig. 5), single factor ANOVA tests were performed. If statistical significance was established with an ANOVA test, we proceeded with t-tests to determine which wires were statistically different from each other. To determine which screws were different from each other, t-tests were performed between each of the screws.

## Results

Dimensional accuracy was confirmed between the virtual model and the drill guides by measurement of the heights and diameters of the drill guides (Table 2). All guides were generated in ScanIP from primitive cylinder of height 120 mm and diameter 40 mm. Calipers used for measuring had a partial surface contact error of ±0.02 mm and a scale shift error of ±0.03 mm for the diameter and a partial surface contact error of ±0.03 mm and a scale shift error of ±0.05 mm for the height [15].

**Table 2:** Confirmation of dimensional accuracy between the computer-generated guide wire model and the 3D printed guides

Guide	Height (mm) (% Error)	Diameter (mm) (% Error)
Percutaneous	120.05 (0.040%)	40.15 (0.38%)
FNS	119.96 (0.030%)	40.08 (0.20%)
DHS	120.13 (0.11%)	40.06 (0.15%)

Figure 4 describes displacement of guide wires relative to their ideal positions. The superior-anterior percutaneous screw (PERC-S1) had an average displacement of 3.38 ± 0.25 mm, the superior-posterior screw (PERC-S2) had an average displacement of 3.17 ± 0.22 mm, and the inferior screw (PERC-S3) had an average displacement of 3.03 ± 0.12 mm. The FNS wire had an average displacement of 1.59 ± 0.18 mm. The DHS wire had an average displacement of 1.03 ± 0.19 mm. All individual displacement values were less than 5 mm.

Figure 5 describes angular deviation of guide wires relative to their ideal positioning. The first percutaneous screw had an average angular deviation of 3.91 ± 0.32^o^, the second had an average angular deviation of 4.17 ± 0.29^o^, and the third had an average angular deviation of 4.21 ± 0.33^o^. FNS had an average angular deviation of 2.81 ± 0.64^o^. DHS had an average displacement of 2.59 ± 0.39^o^. For both we found a statistically higher difference in displacement between each of the percutaneous screw guide wires compared with FNS and DHS guide wires. For angular deviation, we found that DHS was significantly lower than the rest of the screws.

## Discussion

Our custom 3D printed drill guides proved accurate in guiding the placement and angular positioning of guide wires for stabilizing hip fractures. We define accuracy here, relative to existing literature, which we will further describe. We found that both displacement and angular deviation values are consistent with previously published literature examining the accuracy of custom drill guides [11, 16]. Raajmaakers et al. [11] found a maximum angular deviation of 2.9^o^ and displacement of 2.1 mm – comparable to our accuracy for FNS and DHS placement. We did observe that guides with multiple guide wires (PERC) had lower positional accuracy and angular deviation, likely due to shifting of the guide which was compounded with each wire that was placed. This phenomenon of added error also makes the case that added fixation of the guides, such as with a Kirschner wire, would also lead to lower accuracy. This can be improved in the future by refining the technique of use of the drill guide or creating more novel ways to take advantage of local anatomic structures for stability such as the intertrochanteric crest and, in the cadaver model, wrap around the lesser trochanter near the iliopsoas insertion. Creating a multisegmented drill guide, with different modular pieces for each guide wire is also a possible solution.

Although the drill guides did perform accurately, the drill guide for the percutaneous screw systems performed relatively inaccurately when compared with other two drill guides. This is likely because the percutaneous guide required three wires instead of one, and we suspect that the drill guide shifted slightly during each wire insertion. We might have otherwise expected FNS to also be statistically smaller in angular deviation compared to the percutaneous screw guide wires, as DHS was, however there was a larger variation in measurement in angular deviation of FNS which made the difference in angular deviation statistically insignificant.

The iterations of the drill guide designs (Table 1) were useful to learn about the proper characteristics which refined both the strength and accuracy of the guides. Dimensional accuracy was confirmed to be within or near FormLabs stated dimensional tolerances (Table 2) [17]. Significant learning points included structuring the channels in which the guide wires travel through to give them a significant enough length to provide accuracy and stability so that they do not break down during the drilling process (giving way to the turret design in the last two iterations). Equally, using the surrounding anatomy to our advantage, such as the greater trochanter and the metadiaphyseal and diaphyseal shafts to provide a secure point for the guides to attach to were important. Although admittedly a limitation of our method of securing the drill guides on the greater trochanter, as we have done in this study, would likely not work in a patient or cadaveric model. This is because multiple abducting and external rotation muscles attach to the greater trochanter, making guide attachment more challenging. Different shaped tips of the drill guides (Fig. 2) also proved useful in boring through the femur steadily and with enough purchase within the sawbone. The initial threaded guide wire was not sufficient in giving the wire’s path sufficient accuracy (Fig. 2B) compared with the drill-bit tip.

Other models, particularly animal, have attested to the robust nature and feasibility of 3D printed drill guides [12, 13] – defining graded levels of 2 mm intervals of displacement. When compared to guide wire placements in Sakai [14], our measurements are comparable to errors in angular deviation and displacement mentioned in total hip arthroplasty guide wire placement. The advantage of our method, over methods which differ in technique are that 3D printed guides do not require additional training for surgeons and personalized guides are based on the patient anatomy. Surgeons, in fact, can use this method to pre-operatively plan surgeries using the 3D computer model which provides for manipulation of the model and more functional viewing compared with the traditional method of 2D planar fluoroscopy. Although further study is required, we expect that this may be a time-saving solution in the operating room. Nevertheless, such comparisons to these more purely technique-driven solutions gives us confidence that our models are robust enough for cadaveric models and, should they prove successful in cadaveric models, be ready for clinical trials in patients.

## Limitations

Our guide was designed for sawbones, and a more minimalistic design will have to be used for clinical practice. Soft tissue generally would complicate the application of a drill guide. Using CT in conjunction with knowledge of soft tissue attachments would enhance the design and application of drill guides in realistic patient scenarios. Any future drill guide designs for use in cadaveric models or in real patients would require modification of this design, tailored to model/patient specific characteristics such as neck anteversion or retroversion, while maintaining secure attachment to the inferolateral aspect of the greater trochanter.

## Conclusion

The custom 3D printed drill guides provided for a robust accurate application of Percutaneous Screw, FNS, and DHS systems’ guide wires. Drill guides which are used in the placement of more than 1 guide wire were found to have a lower relative positional accuracy. This work, along with the iterations of guides that we have completed, will inform future development of drill guides for use in cadaveric models and, ultimately, personalized and tailored for patients who require femoral neck stabilization.

## Data Availability

The datasets used and/or analyzed during the current study are available from the corresponding author on reasonable request.
