# Correction: Accuracy of guide wire placement for femoral neck stabilization using 3D printed drill guides

**DOI:** 10.1186/s41205-022-00153-9

**Published:** 2022-08-09

**Authors:** Gregory R. Roytman, Alim F. Ramji, Brian Beitler, Brad Yoo, Michael P. Leslie, Michael Baumgaertner, Steven Tommasini, Daniel H. Wiznia

**Affiliations:** 1 Orthopaedics and Rehabilitation, Yale School of Medicine, Yale University, New Haven, CT USA; 2 Yale Center for Medical Informatics, Yale School of Medicine, Yale University, New Haven, CT USA; 3 VA Connecticut Healthcare System, Veterans Health Administration, West Haven, CT USA; 4 Biomedical Engineering, Yale School of Engineering and Applied Science, Yale University, New Haven, CT USA; 5 Mechanical Engineering & Materials Science, Yale School of Engineering and Applied Science, Yale University, New Haven, CT USA

**Correction: 3D Printing in Medicine 8, 19 (2022)**

**https://doi.org/10.1186/s41205-022-00146-8**

Following publication of the original article [1], the authors reported that there was an issue with figures overlapping in Table 1.

**Table 1:**
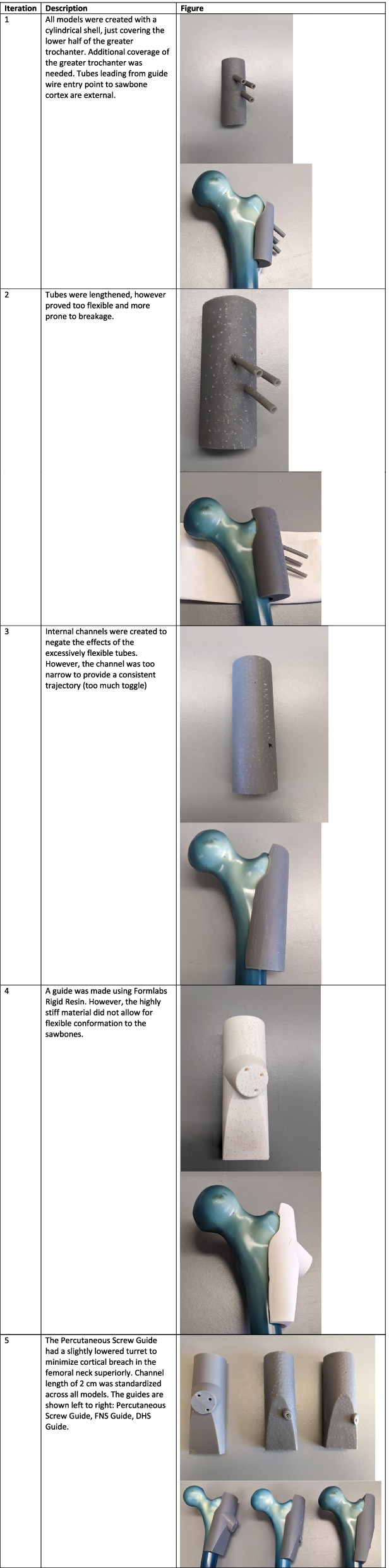
Iterations of 3D printed drill guide being used to drill osteoporotic sawbone femurs

The correct Table 1 has been provided in this Correction.

The original article [1] has been corrected.

## References

[1] Roytman GR, Ramji AF, Beitler B (2022). Accuracy of guide wire placement for femoral neck stabilization using 3D printed drill guides. 3D Print Med.

